# Insect Hsp90 Chaperone Assists Bacillus thuringiensis Cry Toxicity by Enhancing Protoxin Binding to the Receptor and by Protecting Protoxin from Gut Protease Degradation

**DOI:** 10.1128/mBio.02775-19

**Published:** 2019-11-26

**Authors:** Blanca I. García-Gómez, Sayra N. Cano, Erika E. Zagal, Edgar Dantán-Gonzalez, Alejandra Bravo, Mario Soberón

**Affiliations:** a Departamento de Microbiología Molecular, Instituto de Biotecnología, Universidad Nacional Autónoma de México, Cuernavaca, Morelos, México; b Centro de Investigación en Biotecnología, Universidad Autonoma del Estado de Morelos, Cuernavaca, Morelos, México; EPFL

**Keywords:** *Bacillus thuringiensis*, Cry toxins, receptor binding, protein chaperone, protoxins

## Abstract

Bacillus thuringiensis Cry proteins are pore-forming insecticidal toxins with specificity against different crop pests and insect vectors of human diseases. Previous work suggested that the insect host Hsp90 chaperone could be involved in Cry toxin action. Here, we show that the interaction of Cry toxins with insect Hsp90 constitutes a positive loop to enhance the performance of these toxins. Plutella xylostella Hsp90 (PxHsp90) greatly enhanced Cry1Ab or Cry1Ac toxicity when fed together to P. xylostella larvae and also in the less susceptible Spodoptera frugiperda larvae. PxHsp90 bound Cry1Ab and Cry1Ac protoxins in an ATP- and chaperone activity-dependent interaction. The chaperone Hsp90 participates in the correct folding of proteins and may suppress mutations of some client proteins, and we show here that PxHsp90 recovered the toxicity of the Cry1AbG439D protoxin affected in receptor binding, in contrast to the Cry1AbR99E or Cry1AbE129K mutant, affected in oligomerization or membrane insertion, respectively, which showed a slight toxicity improvement. Specifically, PxHsp90 enhanced the binding of Cry1AbG439D protoxin to the cadherin receptor. Furthermore, PxHsp90 protected Cry1A protoxins from degradation by insect midgut proteases. Our data show that PxHsp90 assists Cry1A proteins by enhancing their binding to the receptor and by protecting Cry protoxin from gut protease degradation. Finally, we show that the insect cochaperone protein PxHsp70 also increases the toxicity of Cry1Ac in *P. xylostella* larvae, in contrast to a bacterial GroEL chaperone, which had a marginal effect, indicating that the use of insect chaperones along with Cry toxins could have important biotechnological applications for the improvement of Cry insecticidal activity, resulting in effective control of insect pests.

## Abstract (precis)

Bacillus thuringiensis took advantage of important insect cellular proteins, such as chaperones, involved in maintaining protein homeostasis, to enhance its insecticidal activity. This constitutes a positive loop where the concentrations of Hsp90 and Hsp70 in the gut lumen are likely to increase as midgut cells burst due to Cry1A pore formation action. Hsp90 protects Cry1A protoxin from degradation and enhances receptor binding, resulting in increased toxicity. The effect of insect chaperones on Cry toxicity could have important biotechnological applications to enhance the toxicity of Cry proteins to insect pests, especially those that show low susceptibility to these toxins.

## INTRODUCTION

Different microbial pathogens have acquired the capacity to hijack host cellular functions for their own benefit. Several bacteria produce toxins that interfere with signal transduction to modulate and evade innate immune responses (1, 2), and some others affect actin cytoskeleton assembly to facilitate bacterial adherence to host cells (3), while others make use of vesicular trafficking to target intracellular machinery affecting different cellular functions (4). Bacillus thuringiensis (Bt) is an insect pathogen that produces diverse virulence factors to infect and kill their larval hosts (5). However, among the most important virulence factors produced by Bt are the Cry toxins, which target larval midgut cells by forming oligomeric structures that insert into the cell membrane, forming pores that induce cell bursting by osmotic shock lysis (6). Cry toxins are valuable tools for the control of insect crop pests and insect vectors of human diseases (6). Some *cry* genes, like *cry1Ab* and *cry1Ac*, have been introduced into the genomes of different crops, such as corn, cotton, or soybean, producing transgenic plants that resist insect attack (7, 8). However, some important crop pests show low susceptibility to Cry1A toxins (8, 9).

Intracellular chaperones were first described as heat shock proteins (Hsps) whose expression was induced under stress conditions. Hsp90 is an intracellular molecular chaperone highly conserved from bacteria to vertebrates that could constitute up to 2% of total cellular protein (10, 11). Hsp90 is required for the maturation and maintenance of hundreds of client proteins with important functions in different cellular processes, especially in signal transduction and gene transcription (10, 12). This chaperone interacts as a dimer with its client proteins in an ordered ATP-dependent pathway. Hsp90 cooperates with another ATP-dependent intracellular chaperone, Hsp70, among other cochaperones (10, 11). It has been shown that Hsp70 assists unfolded intermediates by recognizing stretches of hydrophobic residues to fold them into their native state, while Hsp90 assists in later stages of folding or activation (12, 13). Interestingly, it was previously shown that Hsp90 can assist different viruses in their life cycle, by assisting some viral proteins at different stages of infection, such as the hepatitis B virus reverse transcriptase, suggesting that this pathogen makes use of the host Hsp90 protein to facilitate its own replication (14, 15). Also, Hsp90 is required for the efficient transfer of the cholera toxin catalytic subunit from the endoplasmic reticulum to the cytosol, where it disrupts cell homeostasis by altering cellular cAMP levels (16, 17). Regarding Cry toxins active against mosquitoes, it was shown that Aedes aegypti larvae with reduced *hsp90* gene transcript levels, induced by *hsp90* gene silencing (RNA interference [RNAi]), showed 4-fold tolerance to Cry11Aa (18). In the lepidopteran insect Galleria mellonella, inhibition of Hsp90 activity resulted in higher tolerance to Bt infection (19). Finally, proteomic analysis of insect cellular proteins that bind Cry1Ab or Cry1Ac toxins revealed binding of these toxins to Hsp70 in two different lepidopteran species (20, 21), suggesting that Hsp70 and Hsp90 may assist in Cry toxicity.

Bt Cry1A proteins are synthesized as 130-kDa protoxins that upon proteolytic activation by insect gut proteases release a 60-kDa toxic core composed of three domains. Domain I, a seven-α-helix bundle, is implicated in toxin oligomerization, membrane insertion, and pore formation. Domains II and III, mainly composed of β-sheets, are involved in insect specificity by mediating toxin binding to larval gut proteins such as glycosylphosphatidylinositol (GPI)-anchored proteins like aminopeptidase N (APN) or alkaline phosphatase (ALP) or to transmembrane proteins such as cadherin (CAD) or ATP binding cassette transporters (ABCC2) (6, 22). Cry toxins bind sequentially to these insect gut proteins, resulting in toxin oligomerization and pore formation (5, 6). Recent data showed that both forms of Cry proteins, the protoxin and the activated toxin, are able to kill insects, acting via different pathways (23, 24). The C-terminal protoxin domain is composed of four additional domains (25), and it has been shown that this protoxin region is directly involved in binding to APN and ALP receptors, facilitating the further binding of protoxin to the CAD receptor by means of exposed domain II loops (26). The pore formation activity observed after Cry1Ab protoxin bound to the CAD receptor and was activated by membrane proteases is different from the pore formation activity observed after activated toxin bound to CAD (23). The main differences are in the heat sensitivities of these oligomers, their abilities to insert into synthetic membranes, and the pore characteristics (23). Bioassay data confirmed these two independent pathways of toxicity for protoxin or activated toxin since different insect populations resistant to activated Cry1Ac toxin were far more sensitive to the Cry1Ac protoxin (24).

Since Cry toxins burst insect gut cells, releasing the cellular content, it is possible that abundant intracellular proteins could interact with the Cry proteins, influencing their toxicity and forming a positive loop for toxin action. Hsp90 is highly conserved in different organisms and is an abundant cellular protein, and its principal role is to assist protein stability and function (27). Therefore, here, we determined the effect of Hsp90 on Cry1A toxicity, stability, and function.

## RESULTS

### *Plutella xylostella* Hsp90 enhances the toxicity of Cry1Ab and Cry1Ac toxins.

**FIG 1:**
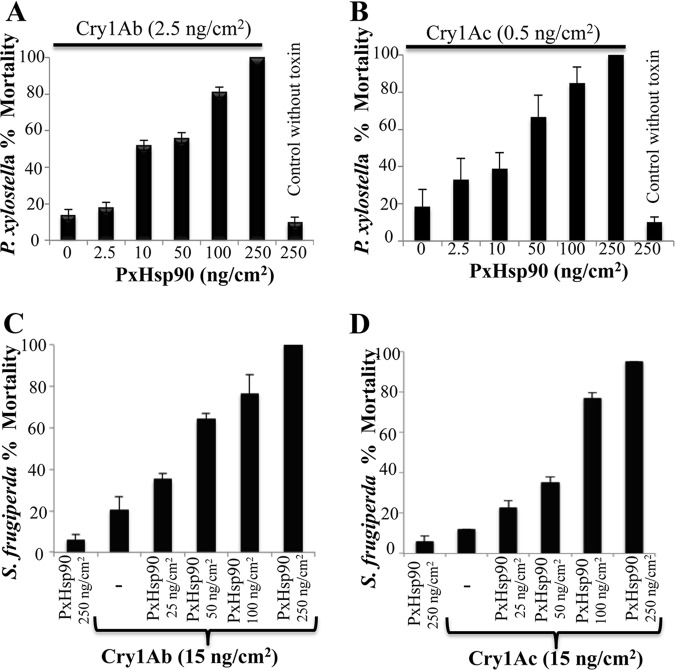
PxHsp90 enhances Cry1Ab and Cry1Ac toxicity against *Plutella xylostella* and Spodoptera frugiperda. (A) Percentage of *P. xylostella* larval mortality after treatment with 2.5 ng/cm^2^ of Cry1Ab protoxin in the presence of increasing concentrations of PxHsp90. (B) Percentage of *P. xylostella* larval mortality after treatment with 0.5 ng/cm^2^ of Cry1Ac protoxin in the presence of increasing concentrations of PxHsp90. The last lanes in panels A and B show mortality rates with 250 ng/cm^2^ of PxHsp90 in the absence of protoxin proteins. Data with standard deviations represent means of results from three treatments using 24 larvae per treatment in each repetition. (C) Percentage of *S. frugiperda* larval mortality after treatment with 15 ng/cm^2^ of Cry1Ab protoxin in the presence of increasing concentrations of PxHsp90. (D) Percentage of *S. frugiperda* larval mortality after treatment with 15 ng/cm^2^ of Cry1Ac protoxin in the presence of increasing concentrations of PxHsp90. Data represent means of results from three experiments performed with 24 larvae per treatment.

The *hsp90* gene from the lepidopteran insect Plutella xylostella was cloned as described in Materials and Methods for P. xylostella Hsp90 (PxHsp90) production in Escherichia coli cells. *P. xylostella* is an important pest of cruciferous crops worldwide; it is highly susceptible to Cry1Ab and Cry1Ac toxins and was the first example of the evolution of insect resistance to these proteins under field conditions (28). To determine the effect of PxHsp90 on Cry1Ab or Cry1Ac toxicity, we performed toxicity bioassays of Cry1A protoxins using a protein concentration that would induce 10% mortality against *P. xylostella* larvae (2.5 ng/cm^2^ of diet for Cry1Ab and 0.5 ng/cm^2^ for Cry1Ac), in the presence of increasing concentrations of PxHsp90. Figure 1 shows that in the presence of PxHsp90, the toxicity of Cry1Ab (Fig. 1A) and Cry1Ac (Fig. 1B) was enhanced in a concentration-dependent manner. In the presence of 50 or 100 ng/cm^2^ of PxHsp90, the toxicity of both Cry1A proteins was enhanced 4- to 8-fold (*P* < 0.0001 for Cry1Ab and *P *< 0.0013 for Cry1Ac, suggesting extremely significant differences), while the presence of 250 ng/cm^2^ of PxHsp90 resulted in toxicity enhancement of up to 100% mortality (*P *< 0.0001 for both toxins). The control of PxHsp90 at the highest concentration (250 ng/cm^2^) was innocuous to the larvae since a low mortality rate (5%) was observed (Fig. 1). Since Spodoptera frugiperda is an important corn pest that is less sensitive to Cry1Ab or Cry1Ac (29) than *P. xylostella*, we determined the effect of PxHsp90 on the toxicity of Cry1Ab or Cry1Ac toxins to this insect pest. Figure S1 in the supplemental material shows the toxicity bioassay results for Cry1Ab and Cry1Ac toxins against an S. frugiperda population from Mexico, showing that they induced 40 to 60% mortality with 150 to 250 ng/cm^2^, indicating very low susceptibility to Cry1A toxins. We analyzed the effect of increasing concentrations of PxHsp90 when mixed with 15 ng/cm^2^ of either Cry1Ab or Cry1Ac. This tested Cry1A concentration shows low mortality rates (5 to 15% after subtracting the mortality rate of the control) for *S. frugiperda*. Figure 1 shows that PxHsp90 increases the toxicity of either Cry1Ab (Fig. 1C) or Cry1Ac (Fig. 1D) protein, reaching 100% mortality when mixed with 250 ng/cm^2^ of PxHsp90 (*P *< 0.0001). The controls of PxHsp90 (250 ng/cm^2^) showed 5% mortality, similar to the mortality observed for a control with just buffer in the diet.

10.1128/mBio.02775-19.1 FIG S1 *S. frugiperda* shows low susceptibility to Cry1Ab or Cry1Ac toxin. (A and B) Percentages of *S. frugiperda* larval mortality after treatment with increasing concentrations of Cry1Ab protoxin (A) or Cry1Ac protoxin (B). C is the control with buffer and without toxin. Data represent the means of results from three experiments performed with 24 larvae per treatment. Download FIG S1, TIF file, 0.3 MB. Copyright © 2019 García-Gómez et al. 2019 García-Gómez et al. This content is distributed under the terms of the Creative Commons Attribution 4.0 International license.

### Binding of PxHsp90 to Cry1A protoxins depends on its chaperone activity.

**FIG 2:**
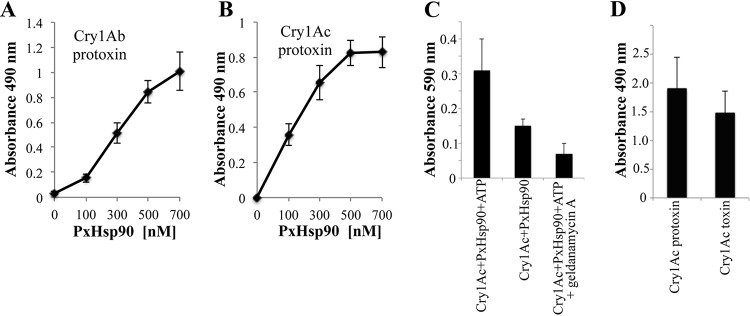
Binding of PxHsp90 to Cry1Ab and Cry1Ac toxins. (A to C) ELISAs of binding of Cry1Ab protoxin (A) or Cry1Ac protoxin (B and C) to PxHsp90. ELISA plates were coated with 0.5 μg of each protoxin, and after washing of unbound proteins, the plates were incubated with different concentrations of PxHsp90, which was revealed with anti-His antibody as described in Materials and Methods. Panel C shows an ELISA of binding of a nonsaturating concentration of PxHsp90 (100 nM) to Cry1Ac with 1 mM ATP (lane 1), without ATP (lane 2), or with 20 μM geldanamycin (Hsp90 inhibitor) containing 1 mM ATP (lane 3). (D) Binding of PxHsp90 (300 nM) to 0.5 μg Cry1Ac protoxin or 0.5 μg activated toxin.

The activity of Hsp90 protein depends on its direct interaction with the client proteins and ATP consumption (10, 11, 27). To analyze the binding of PxHsp90 to Cry1Ab or Cry1Ac toxins, binding enzyme-linked immunosorbent assays (ELISAs) were performed, showing that PxHsp90 bound to both Cry1Ab (Fig. 2A) and Cry1Ac (Fig. 2B) protoxins, in a concentration-dependent way. The chaperone activity of Hsp90 protein relies on the hydrolysis of ATP, and it is inhibited by the inhibitor geldanamycin A, which binds to the ATP binding site in the chaperone and inhibits the Hsp90-mediated conformational maturation/refolding reaction (10, 30). Figure 2C shows that binding of PxHsp90 to Cry1Ac protoxin was significantly reduced in the absence of ATP (Fig. 2C, lane 2). Also, the PxHsp90-Cry1Ac interaction was inhibited in the presence of ATP and geldanamycin A (Fig. 2C, lane 3), indicating that the chaperone activity of PxHsp90 is involved in Cry1Ac binding. Finally, we compared the binding of PxHsp90 to Cry1Ac protoxin and to Cry1Ac activated toxin. Figure 2D shows that PxHsp90 bound similarly to the Cry1Ac protoxin or activated toxin.

### PxHsp90 assists Cry1Ab protoxin in receptor binding.

**FIG 3:**
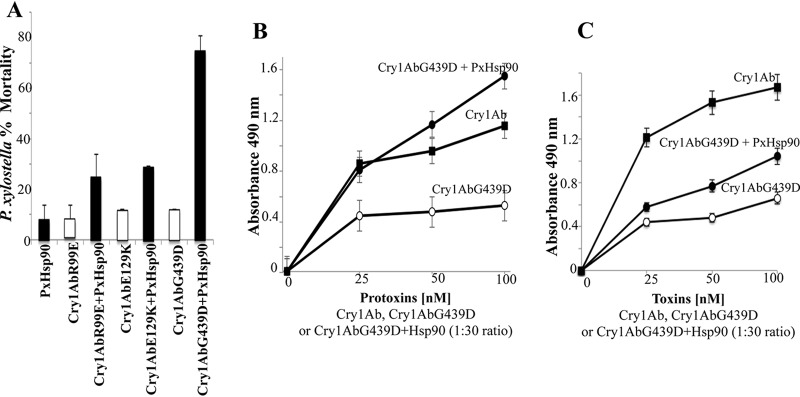
PxHsp90 assists Cry1Ab protoxin in binding to the CAD receptor. (A) *P. xylostella* mortality assays using 35 ng/cm^2^ of Cry1AbR99E, Cry1AbE129K, or Cry1AbG439D protoxin in the absence and presence of 1 μg PxHsp90. Data represent means of results from three replicates of 24 larvae per treatment with standard deviations. (B) ELISAs of binding of Cry1Ab or Cry1AbG439D protoxin and a mixture of Cry1AbG439D protoxin-PxHsp90 at a 1:30 ratio (protoxin to chaperone) to CAD CR12. (C) ELISAs of binding of Cry1Ab or Cry1AbG439D activated toxin and a mixture of Cry1AbG439D activated toxin-PxHsp90 at a 1:30 ratio (toxin to chaperone) to CAD CR12. ELISA plates were coated with 0.5 μg of CR12. After washing, the toxin bound to CR12 was revealed with an anti-Cry1Ab antibody as described in Materials and Methods.

It has been shown in different organisms that Hsp90 can suppress mutations of its client proteins, since reducing the Hsp90 reservoir results in multiple phenotypes depending on the previous silent genetic variation of the client proteins (31, 32). In order to analyze if a particular step of the mode of action of Cry1Ab toxin was assisted by PxHsp90, we analyzed the capacity of PxHsp90 to suppress single point mutations of nontoxic Cry1Ab mutants affected in different steps of its mode of action, like receptor binding (Cry1AbG439D) (33), oligomerization (Cry1AbR99E) (34), or oligomer membrane insertion (Cry1AbE129K) (35). Figure 3A shows that Cry1AbG439D protoxin recovered substantial toxicity in the presence of PxHsp90 (*P* < 0.0001), in contrast to the Cry1AbR99E and Cry1AbE129K mutants, which showed only a slight increase in their toxicity (*P *= 0.0699 and *P *= 0.008, respectively). These results suggest that PxHsp90 assists Cry1Ab protoxin in its binding to receptors. The Cry1AbG439D mutation is located in domain II loop 3, which is involved in receptor binding, such as binding to Manduca sexta CAD repeat 12 (CR12) (26, 33, 36). To analyze the effect of PxHsp90 on the binding of Cry1Ab to its receptors, ELISAs of binding of Cry1AbG439D protoxin or activated toxin to the M. sexta CR12 fragment were performed. Figure 3B shows that in the presence of PxHsp90, the Cry1AbG439D protoxin recovers substantial binding to CR12, similar to the binding of the Cry1Ab protoxin to CAD. Figure 3C shows that in the case of activated toxin, PxHsp90 slightly enhanced the binding of Cry1AbG439D to CAD. The enhancing effect of Hsp90 on the binding of Cry1AbG439D activated toxin to CAD (Fig. 3C) was much lower than that observed for the Cry1AbG439D protoxin (Fig. 3B). These results show that PxHsp90 enhances Cry1A toxicity, at least in part, by assisting in the binding of Cry1Ab protoxin to the CAD receptor.

### PxHsp90 protects Cry1A protoxins from protease degradation.

**FIG 4:**
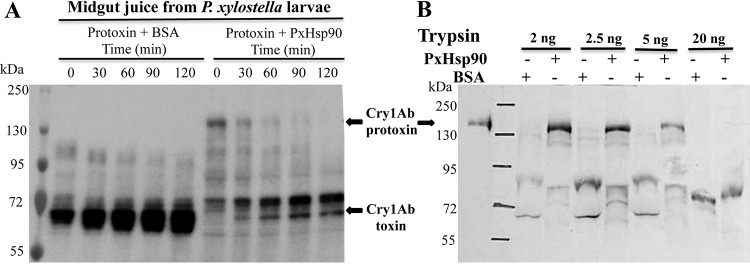
PxHsp90 protects Cry1Ab protoxin from protease degradation. (A) Treatment of Cry1Ab protoxin with midgut juice from *P. xylostella* as described in Materials and Methods. One microgram of protoxin was treated with the gut proteases for different times in the presence of either BSA or PxHsp90 and revealed by Western blotting using the anti-Cry1Ab antibody. (B) Treatment of Cry1Ab protoxin for 1 h with different concentrations of trypsin in the presence of either BSA or PxHsp90. The protoxin was revealed by Western blotting using an anti-Cry1Ab antibody. Control Cry1Ab protoxin without protease treatment is shown in the first lane. Representative results from three replicates are shown. The arrows indicate the positions of the 130-kDa Cry1Ab protoxin and the 60-kDa activated toxin.

For many client proteins, their interaction with the chaperone Hsp70 or Hsp90 prevents or delays their degradation (13, 27). Since PxHsp90 enhanced Cry1Ab protoxin insecticidal toxicity and also binding of protoxin to CAD, we analyzed if the enhancement of Cry1Ab toxicity was related to higher stability of Cry1Ab protoxin against protease treatment. We analyzed the activation of Cry1Ab protoxin with midgut juice proteases isolated from *P. xylostella* larvae in the absence or presence of PxHsp90. In the control sample without a chaperone, bovine serum albumin (BSA) was included at the same concentration as PxHsp90 to differentiate any competition effect that a different protein may have on the protease action. Figure 4A shows Western blot analysis showing that in the presence of PxHsp90, the Cry1Ab 130-kDa protoxin was more stable against treatment with midgut juice proteases since the 130-kDa band of protoxin was still observed after 1 h of treatment. In contrast, in the control treatment, the 130-kDa protoxin was not observed, even with the shortest time of midgut juice incubation. Furthermore, the fully activated 60-kDa toxin band was clearly observed at all times when PxHsp90 was not present in the activation assay, in contrast to when PxHsp90 was present in the assay, which appeared after 60 min of protease treatment (Fig. 4A). Time zero in these assays represents the time needed for mixing Cry1Ab protoxin plus BSA or Cry1Ab protoxin plus Hsp90 with midgut juice and then 3 min of boiling with Laemmli buffer to stop the reaction. The fact that activated toxin was already observed at time zero in the control sample (BSA) indicates that the activation reaction is very fast under these experimental conditions. In the case of the mixture of Cry1Ab protoxin plus Hsp90, the amount of activated toxin at time zero is highly reduced compared to that in the Cry1Ab protoxin-BSA mixture, since the Hsp90 chaperone is present before the addition of midgut juice. The purified protoxin used in these assays in the absence of midgut juice treatment is shown in the first lane of Fig. 4B. These results show that PxHsp90 stabilizes Cry1Ab protoxin by protecting it from midgut protease action. To determine the effect of PxHsp90 on Cry1Ab protoxin stability against other proteases such as trypsin, which is routinely used for *in vitro* activation of Cry toxins, Cry1Ab protoxin in the presence of PxHsp90 or BSA was incubated with different concentrations of trypsin and analyzed by Western blotting. Figure 4B shows that the 130-kDa Cry1Ab protoxin was observed when treated with up to 5 ng of trypsin, in contrast to the control sample with BSA, where Cry1Ab protoxin was degraded at all trypsin concentrations These results show that PxHsp90 also protects against Cry1Ab protoxin degradation by trypsin (Fig. 4B).

### PxHsp70 also enhances the toxicity of Cry1A toxins.

**FIG 5:**
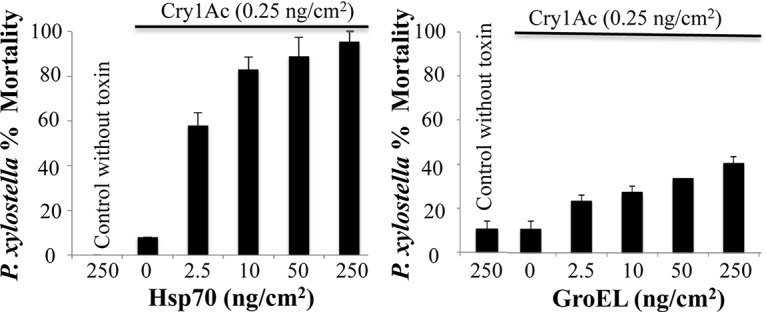
PxHsp70 enhances Cry1Ac toxicity against *Plutella xylostella*. Shown are percentages of mortality of *P. xylostella* larvae treated with 0.5 ng/cm^2^ of Cry1Ac protoxin in the presence of increasing concentrations of PxHsp70 (left) or GroEL (right). Data with standard deviations represent means of results from three treatments using 24 larvae per treatment in each repetition.

To determine if the synergistic effect of the insect PxHsp90 chaperone was specific, we cloned PxHsp70 and also the bacterial GroEL chaperone, from the bacterium Alcaligenes faecalis, which are not related in sequence and mode of action to Hsp90, as described in Materials and Methods. The Hsp70 chaperone binds to unfolded proteins, assisting in protein folding or translocation in an ATP-dependent pathway, while GroEL forms a ring structure that also assists in the folding of denatured proteins coupled to ATP hydrolysis (13, 37). Figure 5 shows that PxHsp70 enhanced Cry1Ab protoxin toxicity to *P. xylostella* larvae in a dose-dependent manner, reaching nearly 100% mortality at 250 ng/cm^2^ of Hsp70 (*P* < 0.0001). In contrast, the GroEL chaperone (250 ng/cm^2^) enhanced the insecticidal activity of Cry1Ac protoxin up to 40% (*P *< 0.0001) (Fig. 5). The stability of these three chaperones against protease digestion with midgut juice proteases was analyzed. Figure S2 shows that GroEL is highly susceptible to degradation by the midgut juice proteases from *P. xylostella*, in contrast to PxHsp90 or PxHsp70.

10.1128/mBio.02775-19.2 FIG S2 PxHsp90 and PxHsp70 are stable against treatment with *P. xylostella* midgut juice, in contrast to GroEL. Shown is Ponceau staining of PVDF membranes (Millipore), with treatments similar to the ones described in the legend of Fig. 4, for mixtures of Cry1Ab protoxin with PxHsp90 (A), PxHsp70 (B), or GroEL (C). The arrows indicate the positions of each chaperone at different time points of protease treatment. Download FIG S2, TIF file, 2.4 MB. Copyright © 2019 García-Gómez et al. 2019 García-Gómez et al. This content is distributed under the terms of the Creative Commons Attribution 4.0 International license.

## DISCUSSION

The Cry toxin receptors are located in the apical microvilli of the midgut epithelial cells, but it has been shown that different Cry toxins could bind, besides membrane receptors, some other intracellular proteins, such as actin, the V-ATP synthase beta subunit, and Hsp70, among others (20, 21). Since Cry proteins exert their toxicity in the plasma membrane by forming lytic pores, the potential role of all these intracellular proteins in the mechanism of action of Cry proteins has been ignored (6). However, gene-silencing experiments showed that Hsp90 was positively involved in Cry11Aa toxicity against the mosquito A. aegypti, while actin was involved in a defense response (18). Here, we show that PxHsp90 binds to Cry1Ab and Cry1Ac depending on the chaperone activity. The chaperone PxHsp90 enhances Cry1A toxicity by two mechanisms, first by assisting in the protoxin receptor interaction but also by stabilizing the protoxin against fast degradation by midgut proteases.

The fact that Hsp90 was able to suppress Cry1AbG439D mutation is compelling evidence that Cry1A toxins could be considered client proteins of this chaperone. It has been shown that the flexibility of Cry1Ab toxin was greatly enhanced at alkaline pH, which is the midgut condition in lepidopteran insects (38). Thus, the midgut conditions could facilitate partial denaturation of Cry protoxin, inhibiting certain steps of its mode of action and enhancing its degradation by gut proteases. Here, we show that PxHsp90 specifically assists Cry1Ab in CAD binding, suggesting that the protoxin could be partially unfolded, diminishing binding to its receptor. It is hypothesized that enhancement of Cry1Ab binding to CAD by PxHsp90 could be due to a stabilizing effect on the Cry1A protoxin structure, such as domain II loop structures optimizing receptor binding. In agreement with this, PxHsp90 had a significantly lower enhancing effect on CAD binding for the Cry1AbG439D activated toxin than for the Cry1AbG439D protoxin (Fig. 3B and C).

Recent work showed that *S. frugiperda* is less sensitive to Cry1Ab since Cry1Ab protoxin is highly unstable against *S. frugiperda* gut proteases and also because Cry1Ab showed a low binding affinity for the receptors located in the brush border membrane (BBM) of *S. frugiperda* midgut cells (39). Here, we show that PxHsp90 also increased Cry1Ab and Cry1Ac toxicity to *S. frugiperda*. It is remarkable that PxHsp90 increases toxicity (100% mortality) at a toxin concentration that shows slight mortality in the *S. frugiperda* population analyzed (Fig. 1C and D). These results show that PxHsp90 is a powerful tool to greatly increase the toxicity of Cry toxins against pests that show low susceptibility to these toxins. The enhancement of Cry1Ab and Cry1Ac toxicity by PxHsp90 in *S. frugiperda* is likely also due to an enhanced stability of protoxin delaying its degradation by midgut juice proteases, as shown here with the midgut juice from *P. xylostella*, and also to an enhanced binding of Cry1A protoxins to receptor proteins present in the BBM of *S. frugiperda*, such as the CAD receptor. This remains to be confirmed.

As mentioned above, Cry1A protoxins are also able to bind to CAD, triggering oligomerization, membrane insertion, and pore formation (23), and a dual mode of action has been proposed for Cry protoxins and activated Cry toxins, based on the different susceptibilities to the activated toxin or protoxin of several resistant populations that were selected with activated Cry toxins (24). Bt plants expressing Cry1Ab or Cry1Ac (7, 8) mainly express activated toxin, probably due to the ability to express a smaller protein in plants. In spite of the proposed dual mode of action of Cry proteins, it has been suggested that protoxin expression in plants would reduce the possibility of the evolution of insect resistance (24). However, protoxins are activated by larval gut proteases, reducing the possibility that complete protoxins reach the midgut epithelium for receptor binding. Recently, it was shown that the C-terminal protoxin fragment of Cry1Ab contains additional binding regions for interaction with ALP and APN, providing a mechanism for protoxin to reach CAD before being completely activated by gut proteases (26). We show here that PxHsp90 stabilizes Cry1Ab protoxin against degradation by midgut proteases and also assists Cry1Ab protoxin in CAD binding, providing additional mechanisms to help the complete Cry1Ab protoxin reach the microvillus membrane for CAD binding. Thus, protection of protoxin degradation increases toxicity, and our data suggest that Hsp90 enhances Cry toxicity by facilitating the toxic pathway exerted by protoxin. As mentioned above, low susceptibility of *S. frugiperda* to Cry1Ab is in part due to the enhanced degradation of the Cry1Ab toxin by gut proteases (39). Thus, stabilizing Cry1Ab protoxin against protease action in insects where low toxicity is in part due to toxin degradation by insect proteases is likely to increase toxicity by stabilizing the protoxin form of Cry proteins.

Analysis of the effect of other chaperones revealed that PxHsp70 was also able to enhance Cry1Ac toxicity similarly to PxHsp90. However, bacterial GroEL is not efficient in enhancing Cry1Ac toxicity unless a very high concentration of GroEL is used in the bioassay (data not shown). However, the lack of a synergistic effect of GroEL on Cry1A toxicity is likely due to its higher susceptibility to degradation by midgut juice proteases, in contrast to that of PxHsp90 or PxHsp70, which is highly stable upon gut protease treatment (see Fig. S2 in the supplemental material). The high stability of PxHsp90 and PxHsp70 against midgut proteases is compatible with their potential role in assisting Cry toxins in the larval gut lumen.

Finally, although we have shown that PxHsp90 is able to increase the toxicity of Cry1A toxins when fed along with the toxin, it still remains to be determined if the intracellular Hsp90 chaperone is secreted out of the cell and what the mechanism is for Hsp90 secretion. We analyzed the protein content of the midgut juice from *P. xylostella* larvae treated or not with Cry1Ac toxin by liquid chromatography-tandem mass spectrometry (LC-MS/MS) analysis, but Hsp90 was not identified (data not shown). Nevertheless, proteomic analysis of the Helicoverpa armigera peritrophic matrix, which separates the alimentary bolus from midgut cells, revealed the presence of Hsp90, indicating that this chaperone is released from the gut cells into the gut lumen and remains associated with peritrophic matrix components (40). Thus, it could be possible that Hsp90 is actively secreted outside midgut cells by an active cellular mechanism, as has been shown to occur in certain tumor cells or under stress conditions (41, 42). However, a simple hypothesis is that the concentration of Hsp90 in the gut lumen is likely to increase as gut cells burst due to the Cry1A pore formation activity creating a positive loop for Cry toxicity. Further experimental evidence is needed to determine if Hsp90 is secreted from *P. xylostella* midgut tissue to the gut lumen. In any case, the effect of PxHsp70 or PxHsp90 on Cry toxicity could have important biotechnological applications to enhance the toxicity of Cry proteins to insect pests that show low susceptibility to Bt toxins.

## MATERIALS AND METHODS

### Expression, purification, and solubilization of Cry1Ab and Cry1Ac protoxins.

The acrystalliferous strain Bt407 *cry* transformed with pHT315-Cry1Ab or the Bt strain HD73 for Cry1Ac production was grown in sporulation medium plates until complete sporulation; in the case of Bt407/pHT315-Cry1Ab, the sporulation medium was supplemented with erythromycin at 10 μg/ml (23, 36). The spores/crystals were harvested by centrifugation and washed three times with washing buffer (0.01% Triton X-100, 50 mM NaCl, 50 mM Tris-HCl [pH 8.5]). For preparing soluble protoxin, crystals were suspended in solubilization buffer (50 mM Na_2_CO_3_ [pH 10.5], 0.02% β-mercaptoethanol) and incubated for 2 h at 37°C. Unsolubilized material was separated by centrifugation, and the protoxin concentration was determined by the Bradford method (43) using BSA as a standard. The quality of soluble protoxins was analyzed by 12% SDS-PAGE.

### Cloning and expression of PxHsp70 and PxHsp90.

Since the Px*hsp90* gene has no introns, in contrast to Px*hsp70*, Px*hsp90* was cloned from whole genomic DNA samples, while Px*hsp70* was cloned from cDNA samples. For Px*hsp70* cloning, total RNA was extracted from 4th-instar *P. xylostella* larvae exposed to 37°C for 3 h. cDNA was constructed from 1.5 μg of the total RNA using reverse transcription-PCR (RT-PCR). The resulting cDNA (3 μl) was used as a template for PCR with specific primers (Table 1) designed based on the known sequence of Px*hsp70* (GenBank accession number JN676213). Oligonucleotides for amplifying the Px*hsp90* gene (GenBank accession number AB214972.1) were designed and used for PCRs using the genomic DNA material from *P. xylostella* larvae as a template (Table 1). PCRs were performed using *Pfu* AccuPrime polymerase and specific primers. The 2.4-kb PCR product for Px*hsp90* was ligated into a pKS EcoRV-digested plasmid, while the 2.0-kb PCR product for Px*hsp70* was ligated into the pJET plasmid. The ligation reactions were used to transform electrocompetent E. coli DH5α. Positive clones were isolated, and DNA was sequenced, confirming the Px*hsp70* or Px*hsp90* sequence. The Px*hsp70* or Px*hsp90* gene was then cloned into the pET28b expression vector after PCR using a pair of oligonucleotides containing the NdeI and BamHI sites (Table 1). Plasmid DNA from positive clones was introduced into E. coli BL21 cells for protein expression. The E. coli BL21-PxHsp90 and BL21-PxHsp70 strains were grown overnight in 2× tryptone yeast (TY) medium supplemented with kanamycin (50 μg/ml), and 2.5 ml of the culture was used to inoculate 250 ml of 2× TY medium supplemented with kanamycin (50 μg/ml). The cultures were incubated at 37°C at 200 rpm to an optical density at 600 nm (OD_600_) of 0.6. Next, 0.5 mM isopropyl-β-d-thiogalactopyranoside (IPTG) was added to induce PxHsp90 or PxHsp70 expression by overnight incubation at 30°C with shaking at 150 rpm. The cells were collected, frozen, and suspended in 1× phosphate-buffered saline (PBS) containing 8 M urea. The samples were sonicated four times for 50 s and centrifuged for 40 min at 48,000 rpm. The supernatants were loaded onto a Ni-nitrilotriacetic acid (NTA)-agarose column, washed with 35 mM imidazole in PBS, and eluted with 250 mM imidazole in PBS with 1 mM ATP and 1 mM Mg to stabilize the proteins. Finally, the PxHsp90 and PxHsp70 samples were dialyzed against the same buffer (1 mM ATP and 1 mM Mg in 1× PBS) using Amicon Ultra 30K filters. These purified proteins were used immediately or kept for only 2 days at −4°C.

**TABLE 1:** Sequences of oligonucleotides

Oligonucleotide	Sequence
ForwGroEL	5′-CATATGACCGCAAAACAAGTTTACTTCG-3′
RevGroEL	5′-GAATTCTTAGAAGCCGCCCATACCACCCATGC-3′
ForwHsp70	5′-CCAGCACATATGGCAACGAAAGCACCC-3′
RevHsp70	5′-CGAGCAGGATCCTTAGTCGACCTCCTCGAT-3′
ForwPxHsp90	5′-ACAATGCCTGAAGAAATGC-3′
RevPxHsp90	5′-GAACTAAATCAGTCTTTGG-3′
RevPxHsp90-BamHI	5′-CGAGCAGGATCCTTAGTCGACCTCCTCCATGCG-3′
ForwPxHsp90-NdeI	5′-CCAGCACATATGCCTGAAGAAATGCAAGCGCAG-3'

### Cloning and expression of GroEL.

Total DNA was extracted from A. faecalis strain MOR02. The *groEL* gene sequence (GenBank accession number KGP00134.1) was obtained from the complete genome sequence of the MOR02 strain (44) (GenBank accession number JQCV0100000024), and specific primers were used to amplify the complete *groEL* gene sequence (Table 1). PCR was carried out under the following conditions: 30 cycles of 30 s at 95°C, 30 s at 56°C, and 1 min at 72°C, followed by a final extension step for 10 min at 72°C, amplified with Phusion DNA polymerase (Thermo Fisher Scientific) in a 50-μl reaction mixture. The purified 1.65-kb reaction product was digested with the NdeI and EcoRI restriction enzymes and inserted into a pET28 cloning vector previously digested with the same enzymes. For the expression of GroEL in E. coli cells, plasmid DNA from a positive clone was transformed into BL21 cells for protein expression, and the protein obtained after induction with IPTG was purified using a Ni-agarose column as described above.

### Effect of PxHsp90, PxHsp70, or GroEL on Cry1A toxicity.

Bioassays were performed with 24 3rd-instar larvae of *P. xylostella* using 24-well plates. The larval diet was surface contaminated with soluble Cry1Ab protoxin (2.5 ng/cm^2^) or soluble Cry1Ac protoxin (0.5 ng/cm^2^ or 0.25 ng/cm^2^) plus different concentrations of the chaperone or protoxin alone without the chaperone. The samples containing the soluble protoxin and chaperone were previously incubated for 30 min at 37°C in PxHsp90 buffer (1 mM Mg and 1mM ATP in 1× PBS). In the case of Cry1Ab mutants, each protein (35 ng/cm^2^) was incubated with PxHsp90 (1.12 μg/cm^2^) and used to contaminate the diet surface in 24-well plates. One larva was placed per well in these plates. Mortality was assessed after 7 days. Each experiment was performed in triplicate (72 larvae per treatment). As a negative control, the diet was surface contaminated with the highest PxHsp90 concentration used without Cry1A protoxin to confirm that PxHsp90 protein was not toxic to the larvae.

In the case of *S. frugiperda*, increasing concentrations of protoxin were used to contaminate the surface of the diet in 24-well plates. For analysis of the effect of PxHsp90 on Cry toxicity, the larval diet was surface contaminated with soluble Cry1Ab protoxin or soluble Cry1Ac protoxin (15 ng/cm^2^) plus different concentrations of the chaperone or protoxin alone without the chaperone. The samples containing the soluble protoxin and chaperone were previously incubated for 30 min at 37°C in PxHsp90 buffer. As described above, the diet was surface contaminated with the highest PxHsp90 concentration used without Cry1A protoxin to confirm that PxHsp90 protein was not toxic to the larvae. One larva was placed per well. These experiments were performed in triplicate. Mortality was assessed after 7 days. For PxHsp70 or GroEL analysis, experiments similar to the ones described above for PxHsp90 were performed.

Data are shown as means ± standard deviations (SD). The *P* value was calculated using Student’s *t* test for two groups (https://www.graphpad.com/quickcalcs/ttest1/), and a *P* value of <0.05 was considered a significant difference.

### Binding of PxHsp90 to Cry1Ab and Cry1Ac protoxins.

The binding of PxHsp90 to Cry1Ab and Cry1Ac protoxins was analyzed by binding ELISAs. A total of 0.5 μg of each protoxin, or activated toxin, in 50 μl of PBS was used to coat ELISA 96-well plates by overnight incubation at 4°C. After the removal of the unbound protoxin, the plate was blocked with blocking buffer (2% skim milk in 1× PBS) for 2 h at 37°C. After the removal of blocking buffer, 50-μl serial dilutions of PxHsp90 in Hsp90 buffer or with 20 μM geldanamycin A (Sigma) were added to the wells and incubated for 1 h at 37°C. The plate was washed three times with washing buffer (1× PBS), 50 μl of anti-polyhistidine-peroxidase-conjugated antibody (1:5,000 dilution) (Sigma-Aldrich) was then added to each well, and plates were incubated for 1 h at 30°C. The reaction was developed with 50 μl of 1 mg/ml *o*-phenylenediamine in substrate buffer (100 mM K_3_PO_4_ [pH 5]) supplemented with 2 μl of hydrogen peroxide. The reaction was stopped by adding 25 μl of 6 N HCl. The plates were read on a microtiter plate reader at an OD_490_. Data represent the means of results from triplicates, and each experiment was repeated two times.

### Effect of PxHsp90 on the binding interaction of Cry1A protoxins with the CAD receptor.

For analysis of binding of Cry1Ab or Cry1AbG439D to CAD, ELISA plates were coated with 0.5 μg of *M. sexta* CAD (CR12) in 100 μl of PBS per well overnight at 4°C. Plates were washed three times with PBS, blocked with 200 μl/well of PBS-M (PBS, 2% skim milk), incubated for 2 h at 37°C, and washed three times with PBS. Different concentrations of Cry1Ab protoxins with or without a 30-fold-higher PxHsp90 concentration (previously incubated with the protoxin during 10 min at 30°C) were added in a 100-μl total volume of PBST (PBS plus 0.1% Tween 20) for 1 h at 37°C. The unbound proteins were removed by three washes with PBST and three washes with PBS. The bound proteins were detected using 100 μl PBST buffer containing anti-Cry1Ab polyclonal antibody (1: 20,000) for 1 h at 37°C. After three washes with PBST and three washes with PBS, we added 100 μl of PBST containing an anti-rabbit-horseradish peroxidase (HRP)-conjugated antibody (1: 20,000) (Santa Cruz Biotechnology) for 1 h at 37°C. Finally, the reaction was developed with the HRP substrates *o*-phenylenediamine and hydrogen peroxide as described above. Each experiment was performed in duplicate, with three repetitions. Statistical calculations (means and standard deviations) and graphics were performed using the Microsoft Excel program.

### Effect of PxHsp90 on Cry1Ac protoxin proteolysis.

*P. xylostella* midgut juice was obtained from dissected midguts from 4th-instar larvae. Briefly, larvae were incubated on ice for 10 min, and midguts were dissected by cutting of the insect cuticle and forceps extraction. Twenty to thirty midguts were collected in a single Eppendorf tube and centrifuged at 1,000 rpm for 1 min. The supernatant was collected, and the total protein concentration was determined by the Bradford method using BSA as a standard. For the analysis of the effect of PxHsp90 on protoxin stability against insect midgut juice treatment, PxHsp90 or BSA, at 120 μg, was incubated with 2.5 μg Cry1Ab protoxin in a final volume of 50 μl of the same PsHsp90 buffer. Each treatment mixture was divided into five tubes (10 μl/tube), and 50 ng of *P. xylostella* midgut juice was added to each tube. Tubes were incubated for different times, as indicated in Fig. 5, at 37°C, and the reactions were stopped by adding 3 μl of 4× Laemmli buffer at different time points and 3 min of sample boiling. Each sample was loaded onto an SDS–10% PAGE gel and transferred onto a polyvinylidene difluoride (PVDF) membrane (Millipore) for Western blot detection. For analysis of the effect of Hsp90 on trypsin treatment of Cry1Ab protoxin, 150 μg of PxHsp90 or BSA was incubated with 5 μg Cry1Ab protoxin in the presence of PxHsp90 buffer in a final volume of 50 μl. Each treatment mixture was divided into five tubes: the first of them was the control without protease, and in the following tubes, 2 μl of a trypsin solution was added at a concentration of 20, 5, 2.5, or 2 ng. Tubes were incubated for 1 h at 37°C, and the reaction was stopped with 3 μl of 4× Laemmli buffer and 3 min of sample boiling. One-half of the volume of each sample was loaded and run on an SDS–10% PAGE gel and transferred onto a PVDF membrane (Millipore) for Western blot detection. For Ponceau staining, membranes were incubated with a 0.5% Ponceau (Sigma-Aldrich) solution in 2% acetic acid for 10 min. Membranes were photographed, washed with distilled water, blocked with 3% nonfat dried skim milk in PBS, and incubated for 1 h at room temperature with an anti-Cry1Ab antibody (1:20,000) in PBS. After washing three times for 15 min with washing buffer (0.05% Tween in 1× PBS), the membrane was incubated with an HRP-conjugated rabbit antibody (Sigma) (1:30,000) for 1 h at room temperature and washed three times in the same way. Detection was performed with SuperSignal chemiluminescence (Pierce) according to the manufacturer’s instructions.
