# Supplementary figures and images for: Insect Hsp90 Chaperone Assists Bacillus thuringiensis Cry Toxicity by Enhancing Protoxin Binding to the Receptor and by Protecting Protoxin from Gut Protease Degradation
Source: mBio. 2019 Nov 26;10(6):e02775-19. doi: 10.1128/mBio.02775-19 (PMC6879724; doi:10.1128/mBio.02775-19)

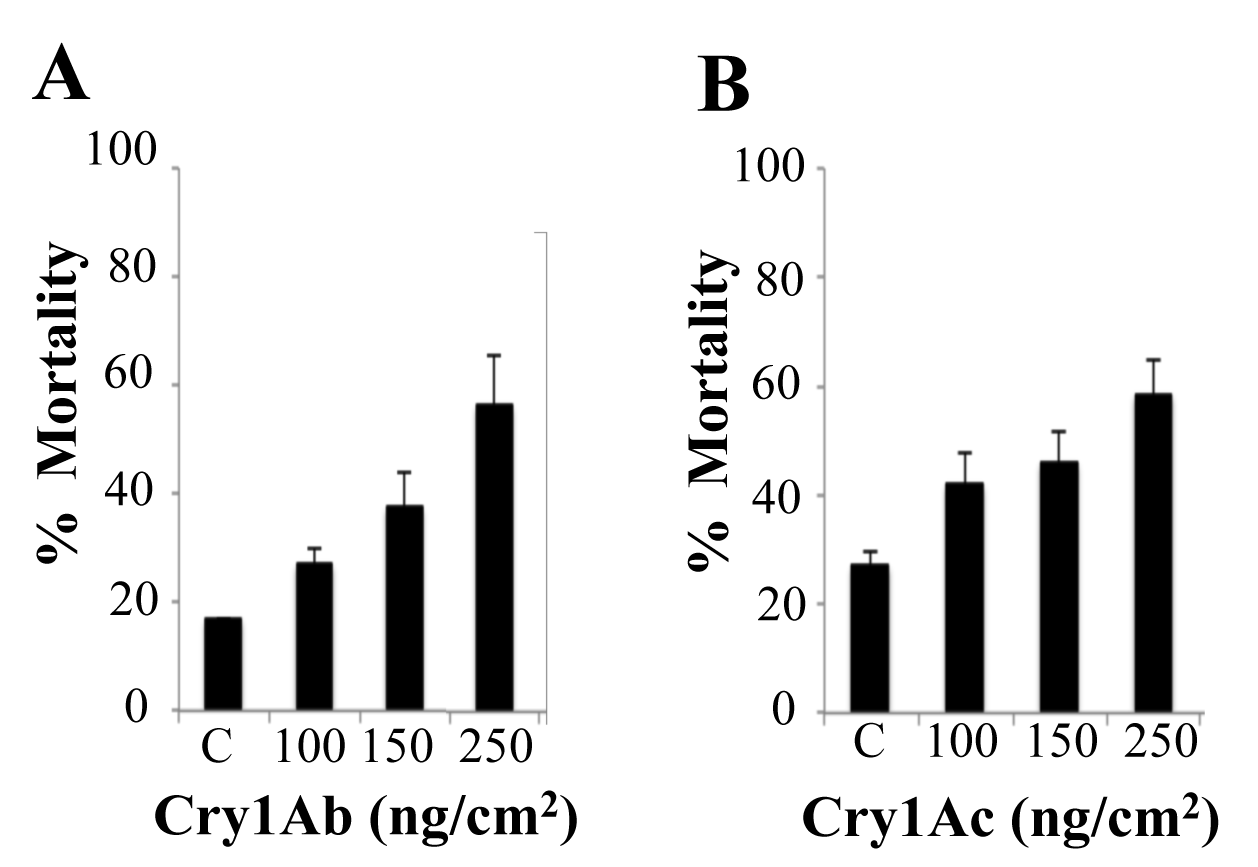

Supplement: FIG S1 [file mBio.02775-19-sf001.tif]

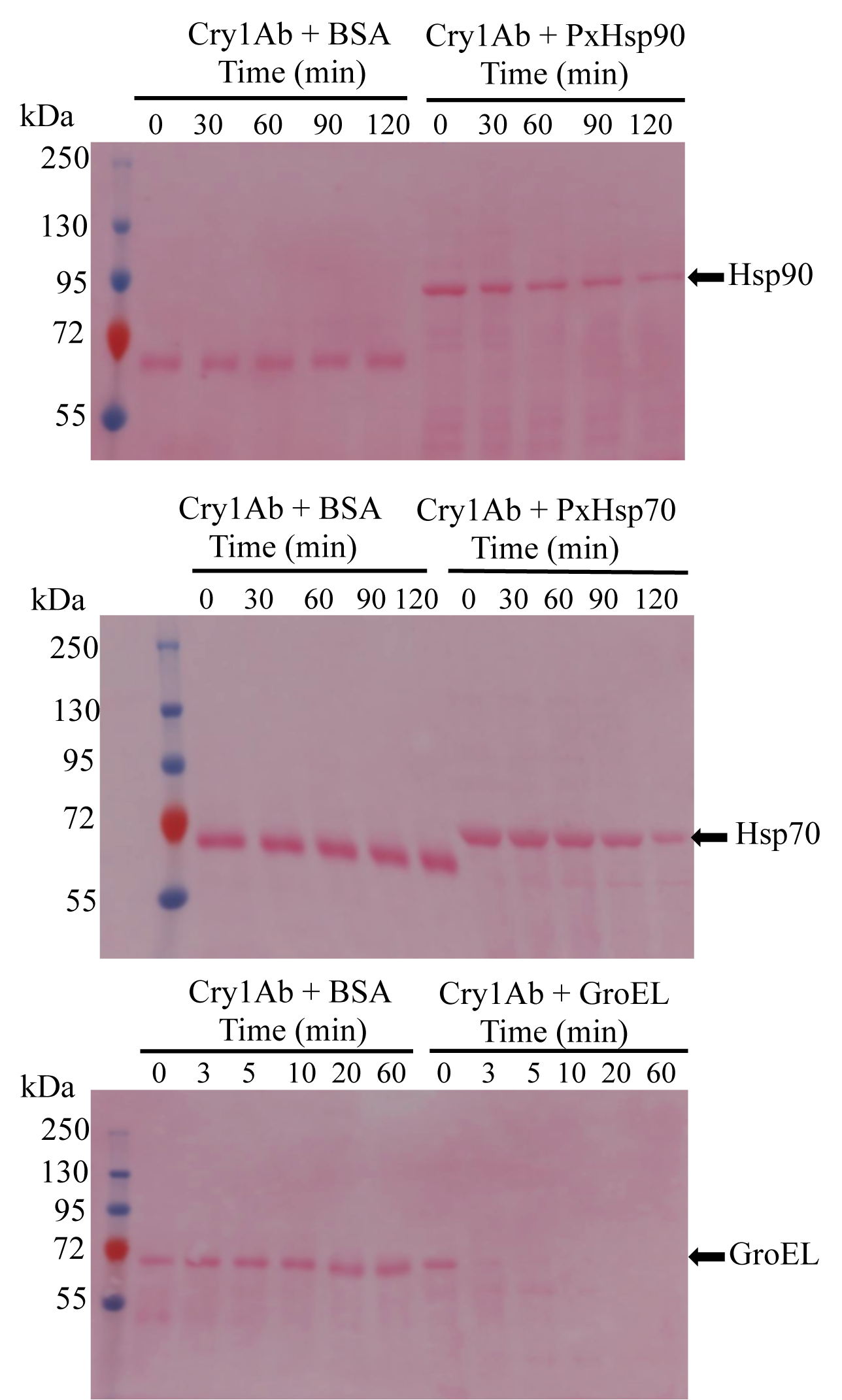

Supplement: FIG S2 [file mBio.02775-19-sf002.tif]
